# Energy balance, leptin, NEFA and IGF-I plasma concentrations and resumption of post partum ovarian activity in swedish red and white breed cows

**DOI:** 10.1186/1751-0147-50-3

**Published:** 2008-01-09

**Authors:** Kristian Konigsson, Giovanni Savoini, Nadia Govoni, Guido Invernizzi, Alberto Prandi, Hans Kindahl, Maria Cristina Veronesi

**Affiliations:** 1 Department of Clinical Sciences, SLU, 75007, Uppsala, Sweden; 2 Dipartimento di Scienze e Tecnologie Veterinarie per la Sicurezza Alimentare, Via Celoria 10, 20133, Milan, Italy; 3 Dipartimento di Morfofisiologia Veterinaria e Produzioni Animali Via Tolara di Sopra 50, 40064 Ozzano Emilia, Italy; 4 Dipartimento di Scienze degli Alimenti, Via Marangoni 97, 33100 Udine, Italy; 5 Dipartimento di Scienze Cliniche Veterinarie, Via Celoria 10, 20133 Milan Italy

## Abstract

In the purpose to provide further information in respect of the relationship between metabolism and post partum (PP) ovarian activity resumption in dairy cows, the aim of the present study was to characterize the energy balance (EB) and leptin, NEFA and IGF-I plasma levels in Swedish Red and White (SRW) cows with and without ovarian activity re-initiation within 7 weeks PP. The study was conducted on 12 primiparous SRW cows fed the same diet as total mixed ration for ad libitum intake. The EB was calculated weekly from parturition until seven weeks PP. Blood samples were collected weekly from one week before until 7 weeks after calving for leptin, NEFA and IGF-I analysis. For progesterone (P4) analysis, blood samples were collected two times per week from parturition until the end of the study. P4 profile was used in addition to the clinical examination to detect cows with and without ovarian activity resumption. The clinical and ultrasonographic examination, coupled with P4 profile analysis showed the resumption of ovarian activity within 7 weeks after calving in 8 (group A) and no ovarian resumption in 4 cows (group B). No significant differences were detected in the whole period of observation in the amount of milk production between the two groups, while the mean milk protein content was significantly lower in group B at the third week PP. The calculated EB was negative in both groups in the first three weeks after calving, but more marked in group B. NEFA and Leptin plasma levels did not show significant differences between the two groups. In conclusion, the results of the present study showed that, when low milk producing primiparous cows are concerned, no significant differences in BW loss, milk yield, EB and leptin and NEFA plasma levels between the cows with and without resumption of ovarian activity within 7 weeks post partum were seen. However, significantly higher IGF-I levels in the first two weeks after calving were found in cows with post partum ovarian activity resumption, highlighting the important role of IGF-I as sensitive signal between metabolism and reproduction.

## Background

After parturition almost every cow experiences a period with high energy requirement related to milk production, frequently associated to an insufficient feed intake. This situation leads to the well known negative energy balance (NEBAL), that seems to be the most important factor affecting the reproductive efficiency after calving. In particular, the resumption of ovarian activity seems to be related to the metabolic status of the cows after calving. However, the interaction between the hypothalamic-pituitary-ovarian axis and the metabolic status of the animal is very complex and not completely explained. Parity has also been considered as an important factor affecting some metabolic, hormonal and reproductive parameters [1,2].

Several hormones and metabolic parameters have been proved to play a role in the relationship between energy balance and post partum reproductive efficiency in dairy cows. Among them, leptin, non-esterified fatty acids (NEFA) and IGF family factors seem to be involved in the re-initiation of ovarian activity in post partum dairy cows.

It has been previously reported [3] that the measurement of circulating IGF-I levels could be used to assess the ability of energy-restricted cows in the resumption of ovarian activity after calving. Huszenicza et al. [4] found higher IGF-I levels in cows with ovulation occurring within 35 days after parturition. Leptin is a peptide hormone produced by white adipose tissue that acts on the hypothalamus as its primary target organ, in particular on regions involved in the regulation of energy metabolism, such as the arcuate, ventromedial, and dorsomedial nuclei of the hypothalamus.

It is also thought to regulate processes that are highly dependent on positive energy supply, such as the onset of puberty, ovarian function, formation of mammary secretory tissue and immune functions. Plasma leptin is also reduced rapidly during periods of undernutrition [5] and this can suggest that it regulate neuroendocrine mechanisms responsible for the partitioning of energy. In well-fed ruminants, central administration of this hormone reduced food intake and energy intake level was positively related to adipose tissue leptin mRNA [6]. In lactating cows has well been established the relationship between plasma leptin, body fatness and feed intake and that the post partum period of negative energy balance such as the delayed resumption of ciclicity is linked to reduced leptin levels [7-9].

The peripartum period is therefore very interesting from a leptin point of view, because of the contemporary occurrence of parturition, lactation and body weight variation. However, several studies found conflicting results concerning the post partum leptin levels in the cow [4,7-10]. Moreover, some studies aimed to clarify the relationship between leptin levels and post partum ovarian activity resumption, showed that there is a tendency in increasing concentrations towards first ovulation [4,9].

Other regulatory mechanisms in the hypothalamus, hypophysis and ovary might play a role in the onset of first postpartum luteal activity [11]. A possibility is that a certain level of hypothalamic sensitivity to leptin has to be reached before GnRH neurons are activated and able to stimulate gonadotrophin secretion from the hypophysis. As leptin also directly stimulates the hypophysis (LH and FSH secretion) and ovary (steroidogenesis), postpartum changes in sensitivity to leptin as a result of high or low leptin receptor expression might also be present in these organs.

NEFA released from lipid stores are either taken up by the udder to provide milk triglycerides or are oxidized in the liver as an alternative energy source. The plasma NEFA concentration is therefore an index of lipid mobilization, with a rise in NEFA pre-partum suggestive of an energy deficit at this time [12,13]. In the liver NEFA can be β-oxidized to obtain acetyl-CoA or esterified to triacylglycerols. In the first case, some of acetyl-CoA produced, in its turn, is oxidized completely to carbon dioxide and the remainder converted to ketone bodies or acetate. Otherwise triacyglycerols can be secreted from liver after incorporation in VLDL (very-low-density lipoproteins) or accumulated in an intracellular lipid droplet.

Despite the well known effect of energy balance on reproduction efficiency in high yielding dairy cows, the effect on medium-low milk cows producers is still unknown. In the purpose to provide further information in respect of the relationship between metabolism and post partum ovarian activity resumption in primiparous dairy cows, the aim of the present study was to characterize the energy balance (EB) and leptin, NEFA and insulin growth factor-I (IGF-I) plasma levels in Swedish Red and White (SRW) cows with and without ovarian activity resumption within 7 weeks after calving.

## Methods

The study was conducted on 12 primiparous SRW cows housed in tie-stall barn. Herd production was about 9000 kg of energy corrected milk/cow/year. All the cows were fed the same diet as total mixed ration for ad libitum intake. Diet composition is reported in Table 1.

**Table 1:** Ingredients and nutrient composition of the total mixed diet fed to primiparous SRW dairy cows

***Ingredients***	***% of DM***
***Mix Silage***	39.00
***Mix Hay***	5.00
***Barley***	18.09
***Oats***	15.12
***Rape Seed Cake***	6.48
***Rape Seed Meal***	2.16
***Brewers Grain***	2.16
***Soybean Meal***	3.78
***Dried Sugar Beet Pulp***	5.40
***Wheat Bran***	0.81
***Mineral Mix***^1^	2.00
***Nutrients analysis***	% of DM
***DM (%)***	52.50
***CP***	17.20
***NDF***	35.94
***Ether extract***	3.72
***Ash***	9.01
***Ca***	0.89
***P***	0.46
***NE_L _MJ/kg of DM***	6.95

^1^Mineral mix: calcium carbonated 40%, sodium bicarbonate 25%, sepiolite 13%, sodium chloride 12%, mono calcium phosphate 4%, yeast 2%, barley 2%, 280,000 IU/kg of vitamin A, 14,000 IU/kg of vitamin D_3_, 1880 mg/kg of vitamin E, 31.50 mg/kg of vitamin B_1_, 21 mg/kg of vitamin B_2_, 11 mg/kg of vitamin B_6_, 0.21 mg/kg of vitamin B_12_, 0.21 mg/kg of biotin, 2100 mg/kg of vitamin PP, 2100 mg/kg of choline, 800 mg/kg of Fe, 700 mg/kg of Mn, 175 mg/kg of Cu, 1750 mg/kg of Zn, 35 mg/kg of I, 7 mg/kg of Co, 11.5 mg/kg of Se.

The energy balance was calculated weekly from parturition until seven weeks after calving from weekly individual measurement of body weight (BW), dry matter intake (DMI), milk production, milk fat, protein and lactose content evaluation and net energy intake (NE_I_; MJ/d) determined by multiplying DMI by the calculated mean net energy for lactation (NE_L_) density of the diet using the following equations [14]:

NE_L _(MJ/kg) = 0.3886936 × Fat % + 0.2288648 × Crude Protein % + 0.165268 × Lactose %

NE_M _= (0.359824 × (0.96 × BW)^0.75^)

NE_G _required for target shrunk weight gain during first lactation values were calculated applying equation from Van Amburgh et al. [15]:

NE_R _= (0.9 × NE_M_) + NE_G _+ NE_L_

EB = NE_I _- NE_R_

where NE_L _= net energy required for lactation, NE_I _= net energy intake, NE_M _= net energy required for maintenance, NE_G _= net energy required for growth, NE_R _= net energy requirement.

Blood samples were collected from the jugular vein in glass tubes with addition of NaHeparin and heparinised blood was immediately centrifuged for 20 minutes at 1000 × *g*. Separated plasma was transferred in plastic tubes and stored at -20°C for leptin, NEFA, IGF-I and progesterone (P4) analysis. Blood samples were collected weekly from one week before until 7 weeks after calving for leptin, NEFA and IGF-I analysis. For P4 analysis, blood samples were collected in the morning, two times per week from parturition until the end of the study.

Progesterone profile was used in addition to the clinical examination to detect cows with and without ovarian activity resumption within the period of observation.

The cows were subjected to a thrice-weekly per rectum palpation of the genital tract supported by the ultrasound examination (real time B-mode linear array scanner with a 7.5 MHz transducer, Aloca SSD-210 DXII) for a better evaluation of ovarian structures morphology and evolution.

Ovarian activity resumption was considered when a series of at least three consecutive samples showed P4 levels ≥ 1 nmol/l, associated to the finding of a corpus luteum at the clinical and ultrasonographic examination. Progesterone was analysed by enhanced luminescence immunoassay (Amerlite, Kodak Clinical Diagnostic Ltd, Amersham, England). Sensitivity of the assay was 0.2 nmol/l. Intra-assay coefficients of variation for 3 control samples (1.5 nmol/l, 17.8 nmol/l and 53.4 nmol/l) assayed in duplicates in 20 assays were 14.9%, 1.9% and 0.6%, respectively. Inter-assay coefficients of variation were below 6%.

Commercially available kits were used to determine leptin (Multispecies Leptin RIA kit; Linco Research, St Louis, MO, USA). As reported by Delavaud et al. [6], the 'multispecies' commercial RIA kit, despite some limitations mainly because of a low sensitivity of the antibody in the low range of leptin values, is as effective and reliable as an ovine-specific RIA in determining leptin plasma profiles in the bovine species. The sensitivity of leptin assay was 0.37 ± 0.01 ng/ml; the intra- and inter-assay coefficients of variation were 4.2 and 8%, respectively. Parallelism with standard curves and scalar dilution of bovine plasma performed for all assays did not show any significant difference [16].

Enzymatic-colorimetric methods were used to determine plasma concentrations of NEFA (Wako Chemicals, Richmond, VA, USA) [16]. IGF-I plasma levels were evaluated by a modified RIA technique [17]. In this method, a cryoprecipitation step was used to eliminate aggregated IGF binding proteins in plasma extracts. Briefly, after acid-ethanol extraction (87.5% ethanol and 12.5% HCl 2 mol/L, v/v), an aliquot of the supernatant was neutralized with 0.855 mol/L Tris base at a ratio of 5:2. The samples were then stored at -20°C for 2 h and immediately centrifuged at 3000 × *g *for 30 min at 4°C. The supernatant was decanted into fresh test tubes and used in the RIA. The recovery of IGF-I added to the plasma was 94.4 ± 3.2%. Rabbit antiserum human IGF-I (GroPep, Adelaide, Australia) has been used. The minimum detectable dose of IGF-I was 1.3 ng/mL. Intra- and interassay coefficients of variation were 8% and 12%, respectively. A goat antirabbit immunoglobulin was used to precipitate the bound hormone.

### Statistical Analyses

The ANOVA of the parameters, body weight variation, milk production, energy balance, leptin, NEFA, IGF-1 plasma level was performed using the MIXED procedure of SAS as repeated measures (SAS/STAT, Version V8, 1999, SAS Inst., Inc., NC, USA)[18]. The model contained the effects of ovarian activity resumption as a series of at least three consecutive samples showed progesterone levels ≥ 1 nmol/l, time (day) after parturition, and their interaction, random effect of animals nested within treatment, and residual error, with individual animals considered the experimental units. The applied model was

Yij = μ + Ti + DJ + (T × D)ij + eij

where Yij = independent variable body weight variation, milk production, energy balance, leptin, NEFA, IGF-1 plasma level; μ = general mean; Ti = effect of the ovarian activity resumption (i = 0,1); DJ = effect of day of sampling; (T × D)ij = effect of the interaction between effect of ovarian activity resumption and time; eij = casual effect of each observation.

## Results

The clinical and ultrasonographic examination, coupled with P4 profile analysis showed the resumption of ovarian activity within 7 weeks after calving in 8 out of 12 cows. Therefore the cows were classified as follows:

Group A (n = 8) with ovarian activity resumption

Group B (n = 4) without ovarian activity resumption.

Mean body weight at week one after parturition was 554 kg for cows of group A and 496 kg for cows of group B and no statistical difference was evidenced, the average weekly body weight losses (kg and %) of the two groups are summarized in Table 2. On average, between the first week after calving and the respective nadir, animals of B group lost 6.46% of BW (32 kg) in comparison with animals of group A that lost 2.5% of BW (14 kg). Percentages of body weight loss were more marked in animals without ovarian activity resumption during all seven weeks after parturition. No statistical differences were detected on body weight loss between the two examined groups.

**Table 2:** Average weekly body weight loss (kg and %) in the two groups of cows compared to calving week

Week after calving		2	3	4	5	6	7	S.E.	P		
									
									ovarian resumption	day	ovarian resumption × day
BW loss (kg)	A	-9	-14	-11	-9	0	-5	8.65	NS	NS	NS
	B	-23	-32	-22	-15	-13	-13				
BW loss (%)	A	-1.56	-2.50	-2.01	-1.65	0.02	-0.9	1.64	NS	NS	NS
	B	-4.64	-6.46	-4.39	-3.08	-2.75	-2.52				

Table 3 includes weekly milk production and milk protein and fat content of cows, average EB values and weekly mean dry matter intake, respectively of group A and B. Average milk production of group A and B during seven weeks was 23.0 kg and 22.8 kg respectively. No statistically significant differences on milk production between the two groups were noted. Milk protein content during the third week of lactation was significantly (P < 0.05) lower in group of cows without ovarian activity resumption in comparison with cows of group A.

**Table 3:** Average weekly milk production, milk composition, DMI and EB in the two groups of cows during the first 7 weeks postpartum

Week after calving		1	2	3	4	5	6	7	S.E.	P		
										
										ovarian resumption	day	ovarian resumption × day
Milk yield (kg/day)	A	17	19	24	23	25	26	27	2.67	NS	*	NS
	B	21	19	20	23	25	25	27				
Milk Fat (%)	A	7.03	5.31	4.53	4.48	4.65	4.41	4.70	0.47	NS	**	NS
	B	7.10	5.40	4.18	4.53	4.53	4.50	4.55				
Milk Protein (%)	A	3.53	3.83	3.78^a^	3.34	3.15	3.20	3.18	0.17	*	*	NS
	B	3.60	3.37	3.25^b^	3.28	3.08	3.23	3.03				
DMI (kg/day)	A	11.3	14.5	15.5	15.8	18.0	17.9	19.3	1.28	NS	**	NS
	B	11.2	11.8	13.6	16.9	17.1	17.5	18.6				
Energy Balance (MJ/day)	A	-28.66	-7.64	-12.66	-3.81	2.65	1.52	5.80	8.31	NS	**	NS
	B	-42.60	-27.48	-2.40	8.19	3.90	4.31	5.71				

^a,b^,* P – value < 0.05.

** P – value < 0.01

Mean values of the B group animals, even if not supported by a statistical evidence, showed a deeper negative EB than animals of group A. While in B group EB was negative in the first three weeks of lactation, in group A a slight NEBAL persisted until the fourth week postpartum. The nadir in both groups was reached in the first week after parturition with -28.66 MJ/day and -42.60 MJ/day for group A and B respectively. EB values between the two groups during all the considered period showed no statistically significant differences.

Weekly mean dry matter intake between two groups was not significantly different in the second and third week of lactation, when animals of group without ovarian activity ingested 2.67 kg and 1.92 kg of dry matter less than group with ovarian activity resumption.

Weekly average NEFA, IGF-I and leptin plasma levels in cows grouped according to their ovarian activity from one week before parturition until seven weeks postpartum are presented in Table 4. NEFA plasma content of both groups reached summit during the first week after calving. Higher values for B group compared to A group (328 vs 299 and 319 vs 255 μEq/l) in the first and the second post partum weeks are not supported by statistical evidence. IGF-I plasma concentration of animals without ovarian activity resumption was lower during all the investigated weeks than values in plasma of animals with ovarian activity resumption; during the first and the second week of lactation the differences between the two groups were statistically confirmed (P < 0.05).

**Table 4:** Average NEFA, IGF-I and leptin plasma levels in the two groups of cows during the period of observation

Week from calving		-1	1	2	3	4	5	6	7	S.E.	P		
											
											ovarian resumption	day	ovarian resumption × day
NEFA (mEq/l)	A	155	299	255	244	214	236	144	134	44.11	NS	**	NS
	B	119	328	319	138	142	134	160	130				
IGF-I (ng/ml)	A	128	86^a^	66^a^	94	69	90	86	86	15.1	**	**	NS
	B	106	37^b^	19^b^	56	42	52	57	52				
Leptin (ng/ml)	A	3.5	3.2	3.4	3.6	3.4	3.3	3.4	3.4	0.43	NS	NS	NS
	B	3.3	3.5	4.4	4.2	4.4	4.3	3.8	3.8				

^a,b ^P – value < 0.05.

** P – value < 0.01

Leptin plasma levels showed no significant differences between cows with (group A) and without (group B) resumed ovarian activity. The levels were relatively stable in group A and slightly fluctuating, and a bit higher, in group B.

## Discussion

The present study was aimed to characterize the features of BW loss, milk production, EB and some hormonal profiles in SRW primiparous cows within 7 weeks after calving. Since the effect of negative EB on reproductive performances in high yielding dairy cows is well known [19-23], in the present study the interest was focused on a breed with a typical medium-low milk production, in order to evaluate the extent of the negative EB and the subsequent effects on reproduction. In addition, since a difference in the adaptation to post partum negative EB has been reported [1,2,24] in primiparous compared to multiparous cows, the study was performed on only primiparous cows.

Buckley et al. [25] and Roche et al. [26] underlined that BW variation during postpartum period has an important role on reproductive performance; a more pronounced loss of BW was observed in the cows that did not resume ovarian activity compared to the cows with ovarian activity resumption within seven weeks postpartum, but the statistical analysis did not evidence differences. It should be considered that in this trial, the most pronounced mean body weight loss (-6.46%) was less than values reported by Heinonen et al. [27] who observed lower reproductive performance in cows that lost more than 10% of BW postcalving compared with cows that lost less than 10%. No significant differences were detected in the whole period of observation in the amount of milk production between the two groups, with the highest (27 kg/day) average production recorded in both groups at the last week of study. These results are consistent with other studies that found no relationship between milk production and reproduction [28,29]. However, most of the recent studies have found a negative relationship between milk production and several fertility traits [22,30-32]. On the contrary, Buckley et al. [25] observed a positive association between milk yield variables and reproductive efficiency.

Milk protein content or milk protein:fat ratio is often used as an indicator of energy balance [27]. The mean milk protein content in animals of group without ovarian resumption during the seven investigated weeks was lower than the protein level of milk produced by A group cows, attaining a statistical significance (P < 0.05) at the third week postpartum. These results can indicate that milk protein content and days to nadir milk protein content can also be indicative of reproductive performance. Fulkerson et al. [33] found severest and prolonged NEBAL in cows with the lowest milk protein content (2.89%), compared with cows with a milk protein content of 3.10%. No differences in milk fat content were observed between the two groups.

The timing of the negative EB nadir has been implicated in the timing of first ovulation [22,23] that occurs on average 30 days postpartum [19,20]. From a number of studies, NEBAL during the first 3 weeks of lactation is highly correlated to the interval to first ovulation [22,23]. The severity and duration of NEBAL is primarily related to dry matter intake and rate of increase during early lactation [20,28]. The calculated EB was negative in both groups in the first three weeks after calving, but more marked in cows without ovarian activity resumption. However, no significant differences between the two groups were evidenced. It should be kept in mind that in this trial EB estimates were based on prediction equations [14] therefore less accurate than an actual experimental determination would have been.

No significant differences between the two groups were evidenced when leptin plasma concentrations were considered. The typical decrease associated to start of lactation, previously described by Accorsi et al [16], was observed. This could therefore suggest that none of the cows experienced a real negative energy balance or, at least, that the scarce body fat loss observed in the group B is not however sufficient to determine a significant leptin levels change. The comparison between the two groups in the NEFA plasma concentrations did not show any significant differences and the levels were always lower than 350 μEq/l. Accorsi et al [16] reported high NEFA levels (around 300–500 μEq/l) in the first 10 days after calving, negatively correlated to leptin, as a sign of marked mobilization from the adipose tissue. This could be considered as a further evidence that, in the present study, none of the cows underwent a strong metabolic variation. The lack of significant differences in BW loss, milk production, EB, and in leptin and NEFA plasma levels between cows with and without ovarian resumption within 7 weeks after calving, seems to suggest that the balance between energy loss and intake was not responsible for the outcome of ovarian resumption. On the other hand, the significant IGF-I differences recorded during the first and the second weeks highlight the important role of the growth factors family in the regulation of reproductive axis, and especially in the follicular growth, as previously reported by Kadokawa et al [34]. As reported in the study of Wathes et al [2] IGF-I plasma concentration can vary depending on the age of animals, as young animals show a higher level. This explains different data from conflicting previous investigations [1,35-37]. In our study, IGF-I plasma concentrations are in agreement with those found by Taylor et al [35] and Wathes et al [2]. Meikle et al [1] found IGF-I and leptin as the best signals between the EB and reproductive performances in dairy cows and reported a more marked decrease in IGF-I plasma levels in primiparous compared to pluriparous cows. The relation between high IGF-I levels and good reproductive performance was also previously reported by Butler [23], with a negative relationship between IGF-I and the interval between calving and the resumption of ovarian cyclicity. The results from the present study seem to stress even more the important role of IGF-I as an extremely sensitive signal between metabolism and reproduction. In fact, despite the absence of other metabolic differences, only IGF-I levels were higher in cows with compared to cows without ovarian activity resumption.

## Conclusion

The results of the present study showed that, when low milk producing primiparous cows are concerned, no significant differences in BW loss, milk yield, EB and leptin and NEFA plasma levels between the cows with and without resumption of ovarian activity within 7 weeks post partum were seen. However, higher significant IGF-I levels in the first two weeks after calving were found in cows with post partum ovarian activity resumption, highlighting the important role of IGF-I as sensitive signal between metabolism and reproduction.

## Authors' contributions

KK and MV carried out the clinical trial, GS took care of energy balance evaluation, NG carried out the NEFA and leptin analysis, GI carried out the statistical analysis, AP performed the IGF-I analysis, and HK designed the study and coordinate the work. All the authors contributed to manuscript draft and results discussion.
